# Knockdown of vascular cell adhesion molecule 1 impedes transforming growth factor beta 1-mediated proliferation, migration, and invasion of endometriotic cyst stromal cells

**DOI:** 10.1186/s12958-019-0512-9

**Published:** 2019-08-23

**Authors:** Juan Zhang, Hui Li, Dan Yi, Chuntian Lai, Haiyan Wang, Wenda Zou, Bei Cao

**Affiliations:** Reproductive Medicine Center, Zhuzhou Central Hospital, No. 166 South Changjiang Road, Zhuzhou, 411200 Hunan Province China

**Keywords:** Endometriosis, Transforming growth factor beta 1, Vascular cell adhesion molecule 1, Proliferation

## Abstract

**Purpose:**

Endometriosis is one of the most common, difficult, and complicated gynecological disorders. Vascular cell adhesion molecule 1 (VCAM-1) has been reported to be aberrantly expressed in patients with endometriosis. However, the exact role and mechanism of VCAM-1 in endometriosis remains unclear.

**Methods:**

The expression of transforming growth factor beta 1 (TGF-β1) and VCAM-1 was determined by quantitative real-time polymerase chain reaction and western blotting. Human endometriotic cells were cultured and their responsiveness to TGF-β1 was evaluated by Cell Counting Kit-8, 5-ethynyl-2′-deoxyuridine, and transwell migration and invasion assays.

**Results:**

The levels of TGF-β1 and VCAM-1 mRNA were upregulated in the endometriotic tissues. Knockdown of TGF-β1 in endometriotic cyst stromal cells caused a marked inhibition of cell proliferation, migration, and invasion. Treatment of endometriotic cyst stromal cells with TGF-β1 resulted in an obvious promotion of cell proliferation, migration, and invasion, and strikingly increased the protein expression of VCAM-1. Silencing of Smad3 abated TGF-β1-stimulated VCAM-1 expression. Furthermore, the promoting effects of TGF-β1 on the proliferation, migration, and invasion of endometriotic cyst stromal cells were blocked by silencing of VCAM-1.

**Conclusion:**

Knockdown of VCAM-1 impedes TGF-β1-mediated proliferation, migration, and invasion of endometrial cells, thereby indicating that VCAM-1 may serve as a therapeutic target for endometriosis.

## Introduction

Endometriosis is considered as one of the most common and complicated diseases in gynecology. Globally, it affected about 10.8 million women in 2015 [1]. The presence, growth, and invasion of functional endometrial glandular epithelium and stroma outside the uterine cavity are hallmark features of endometriosis [2]. Endometriosis can be divided schematically into the following stages: shedding of cells, cell survival, escape from immune surveillance, adhesion to the peritoneum, angiogenesis, and bleeding. Usually, endometriosis develops in the ovaries, fallopian tubes, and tissue around the uterus and ovaries. Rarely, endometriosis occurs in other parts of the body, such as the lung, brain, and skin. Although endometriosis is a benign disease, it shares similarity with other malignancies in characteristics, such as invasion, growth, and high recurrence [3]. Typical clinical manifestations of endometriosis include dysmenorrhea, dyspareunia, pelvic pain, and infertility, which remarkably influence the quality of life of patients with endometriosis [4, 5]. Retrograde menstruation, environmental toxins, müllerianosis, aberrant stem cell function, coelomic metaplasia, and autoimmune have been reported as important contributors to endometriosis [6–10]. Currently, pain medication, hormonal treatment, and surgery are the major therapeutic methods for endometriosis. However, these treatments can improve symptoms, but cannot cure endometriosis. Hence, there is an urgent need to develop novel and effective approaches for endometriosis therapy.

Transforming growth factor beta 1 (TGF-β1), located on chromosome 19q3, is a polypeptide member of the TGF-β superfamily of cytokines [11]. Mature TGF-β1 is composed of 112 amino acids crosslinked by disulfide bonds. TGF-β1 plays a crucial role in a wide variety of cellular processes, such as cell proliferation, differentiation, adhesion, and apoptosis [12]. Under normal conditions, TGF-β1 exists in an inactive state and serves as part of a latent complex consisting of latency-associated peptide (LAP) and latent TGF-β binding protein. Once activated via the proteolytic action of proteinases or the interaction between LAP and integrin αvβ3, αvβ5, αvβ6, or αvβ8, TGF-β1 interacts with its receptors (type I and II: TGF-βRI and TGF-βRII) [13]. Binding of TGF-β1 to TGF-βRII recruits TGFβ-RI to form a transmembrane heterodimer and thereby promotes the activation of TGF-βRI. TGFβ-RI activates the intracellular Smad signaling system and in turn regulates the expression of the TGF-β1-responsive genes, which are involved in cell proliferation, motility, invasion, and metastasis [14, 15]. Increasing attention has been focused on TGF-β1 due to its role in numerous diseases, including endometriosis. To date, however, the molecular mechanism by which TGF-β1 contributes to the development of endometriosis remains poorly defined.

In this study, we aimed to investigate the functional role of vascular cell adhesion molecule 1 (VCAM-1) in TGF-β1-mediated endometriosis in vitro. Our results revealed that knockdown of VCAM-1 impedes TGF-β1-mediated proliferation, migration, and invasion of endometriotic cyst stromal cells, suggesting that VCAM-1 may serve as a promising therapeutic target for endometriosis.

## Materials and methods

### Clinical tissue specimen

Endometriotic tissues were collected from 17 patients with endometriosis and none of them had received any prior hormonal therapy. Endometriosis were diagnosed by laparoscopy and histopathological examination. The mean age of patients with endometriosis was 29 (20–35) years. Normal endometrial tissues were procured from 17 endometriosis-free women undergoing laparoscopy examination for prolapsed uterus or ovarian cyst. These women in the control group have no endometriotic lesions, pelvic adhesions, and infertility. The mean age of women in the control group was 31 (22–39) years. This study was approved by the Ethics Committee at Zhuzhou Central Hospital and written informed consent was obtained from each patient prior to surgery.

### Isolation of endometriotic cyst stromal cells

After excision, the human endometriotic tissue was rinsed with phosphate buffered saline (PBS). The endometrial lining was detached from the myometrium, minced in Hank’s balanced salt solution, and then digested with collagenase II (Solarbio, Beijing, China) at 37 °C for 2 h. Follwing this, they were centrifuged, rinsed, and resuspended in Dulbecco’s modified Eagle’s medium (DMEM)/F12 medium (Solarbio) containing 10% fetal bovine serum (FBS; Solarbio), 100 U/mL penicillin (Solarbio), and 100 mg/mL streptomycin (Solarbio). The cell suspension was then filtered through a 100 mm cell strainer (BD Falcon, Franklin Lakes, NJ, USA). Following this, cells were centrifuged, resuspended, and cultured in DMEM medium in a 5% CO_2_ incubator at 37 °C. At confluence, endometriotic cyst stromal cells were treated with 10 ng/mL recombinant human TGF-β1 (catalog number: PHG9214; Invitrogen, Carisbad, CA, USA) for 48 h.

### Quantitative real-time polymerase chain reaction (qRT-PCR)

Total RNA was isolated from tissues and cells using TRIzol reagent (Invitrogen) in keeping with the manufacturer’s instructions. The quality of RNA was assessed using a spectrophotometer (Thermo Fisher Scientific, Wilmington, DE, USA). A total of 1 μg of RNA was converted to cDNA using SuperScript First-Stand Synthesis System (Invitrogen). qRT-PCR was carried out in triplicate on an ABI Prism 7900 HT Sequence Detection System (Applied Biosystems, Warrington, UK) using the FastStart Universal SYBR Green Master (Roche, Penzberg Germany). The gene-specific primers used in this study were as follows: TGF-β1: forward, 5′-GGA CAT CAA CGG GTT CAC TA-3′ and reverse, 5′-GCC ATG AGA AGC AGG AAA G-3′; VCAM-1: forward, 5′-ACA CCT CCC CCA AGA ATA CAG-3′ and reverse, 5′-GCT CAT CCT CAA CAC CCA CAG-3′; β-actin: forward, 5′-AGG GGC CGG ACT CGT CAT ACT-3′ and reverse, 5′-GGC GGC ACC ACC ATG TAC CCT-3′. Gene expression levels were standardized to the levels of β-actin using the 2^–ΔΔCt^ method. The experiment was independently repeated at least three times.

### Western blot analysis

Total protein was extracted from tissues and cells using RIPA buffer and the quality of protein was evaluated using BCA protein assay kit (Pierce, Rockford, IL, USA). The extracted proteins were resolved on 14% sodium dodecyl sulfate-polyacrylamide gels and transferred onto polyvinylidene fluoride membranes. Thereafter, the membranes were blocked with 5% fat-free milk, incubated with primary antibodies against TGF-β1 (catalog number: ab92486; Abcam, Cambridge, MA, USA), VCAM-1 (catalog number: ab174279; Abcam), Smad3 (catalog number: ab122028; Abcam), and β-actin (catalog number: BM0627; Boster, Wuhan, China) overnight at 4 °C, followed by incubation with horseradish peroxidase-conjugated secondary antibodies (catalog number: BA1050; Boster) for 1 h at 25 °C. Bands were developed using a BeyoECL Moon kit (Beyotime Biotechnology, Shanghai, China) for visualization. The experiment was independently repeated at least three times.

### Cell transfection

To knockdown TGF-β1 or VCAM-1 expression, small interfering RNAs (siRNAs) against TGF-β1 (si-TGF-β1), VCAM-1 (si-VCAM-1),Smad3 (si-Smad3), and a negative control siRNA (si-NC) were designed and synthesized by Sangon Biotech (Shanghai, China). Cell transfection was carried out using Lipofectamine 2000 (Invitrogen) as per the manufacturer’s specifications.

### Detection of cell proliferation capacity

The proliferation of endometriotic cyst stromal cells was determined using Cell Counting Kit-8 (CCK-8) and 5-ethynyl-2′-deoxyuridine (EdU) assays.

For CCK-8 assay, after treatment, endometriotic cyst stromal cells in the exponential growth phase were collected and plated on 96-multiwell plates, followed by incubation with CCK-8 reagent (10 μl; Dojindo, Kyushu, Japan) for 2 h. The absorbance at 450 nm was measured using a spectrophotometer. This experiment was performed with triplicate wells and independently repeated at least three times.

For EdU assay, endometriotic cyst stromal cells were collected after treatment. Cells were seeded on a 24-well plate and incubated with EdU (Beyotime Biotechnology) for 2 h at 37 °C in a 5% CO_2_ incubator. Thereafter, cells were fixed with 4% formaldehyde for 15 min, rinsed with PBS, and permeated with 0.3% Triton X-100 for 15 min. After washing, cells were treated with Click additive solution for 30 min in darkness, followed by staining with Hoechst 33342 for 3 min away from light. The number of EdU-positive cells was counted under a fluorescent microscope (Olympus, Tokyo, Japan). The experiment was independently repeated at least three times.

### Transwell migration and invasion assays

Transwell migration and invasion assays were performed to assess the migration and invasion of endometriotic cyst stromal cells, respectively. For the migration assay, endometriotic cyst stromal cells were resuspended in serum-free medium and then loaded into the upper chambers. The lower chambers were filled with DMEM plus 5% FBS. For the invasion assay, transwell chambers were pre-coated with or Matrigel (BD Biosciences, San Jose, CA, USA). After 48 h of incubation, non-migrated or non-invaded cells on the inner side were removed with a cotton swab. Migrated or invaded cells on the outer side were fixed with 4% paraformaldehyde, stained with 6-diamidino-2-phenylindole (Solarbio), and counted in five representative fields under a light microscope (Olympus). The experiments were performed in triplicate. The experiment was independently repeated at least three times.

### Statistical analysis

Data analyses were carried out using SPSS 20.0 software (SPSS Inc., Chicago, IL, USA). All data were expressed as mean ± standard error of the mean. Statistical comparisons were made by Student’s *t*-test or one-way analysis of variance. The limit of statistical significance was defined as *P* < 0.05.

## Results

### TGF-β1 and VCAM-1 mRNA levels are upregulated in human endometriotic tissues

**Fig. 1:**
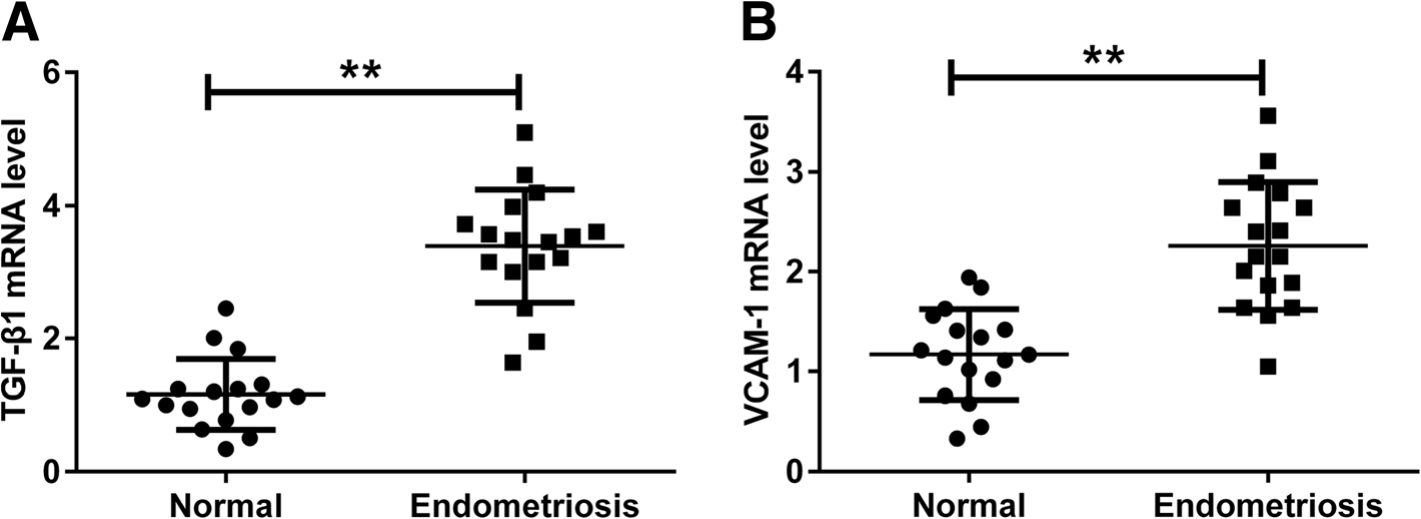
TGF-β1 and VCAM-1 mRNA levels are upregulated in human endometriotic tissues. qRT-PCR analysis of TGF-β1 (**a**) and VCAM-1 (**b**) mRNA expression in human endometriotic tissues and normal endometrial tissues. **P < 0.01

Firstly, we determined the mRNA levels of TGF-β1 and VCAM-1 in human endometriotic tissues and normal endometrial tissues using qRT-PCR. Our results demonstrated that the mRNA level of TGF-β1 was higher in human endometriotic tissues than that in normal endometrial tissues (Fig. 1a). Similarly, VCAM-1 mRNA level was also increased in human endometriotic tissues as compared to that in normal endometrial tissues (Fig. 1b).

### Silencing of TGF-β1 inhibits the proliferation of endometriotic cyst stromal cells

**Fig. 2:**
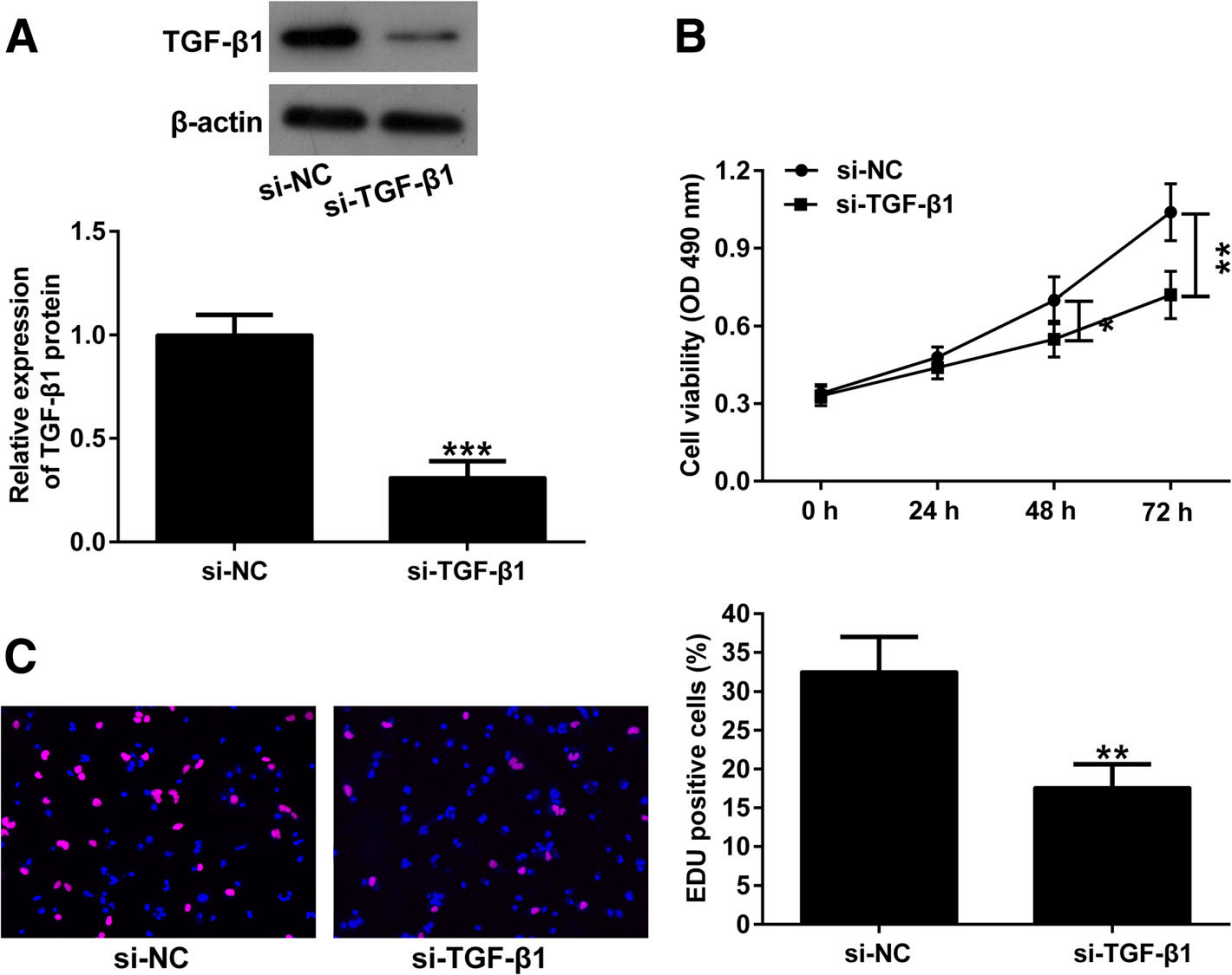
Silencing of TGF-β1 inhibits the proliferation of endometriotic cyst stromal cells. **a** Western blot analysis of TGF-β1 expression showed the reduced protein expression level of TGF-β1 in si-TGF-β1-transfected endometriotic cyst stromal cells. Endometriotic cyst stromal cells were transfected with si-TGF-β1 or si-NC, followed by CCK-8 assay (**b**) and EdU assay (**c**). *P < 0.05, **P < 0.01, and **P < 0.001

Since TGF-β1 mRNA level was upregulated in human endometriotic tissues, we knocked down TGF-β1 expression in endometriotic cyst stromal cells to investigate its functional roles. Western blot analysis revealed that the protein expression of TGF-β1 was strikingly decreased in the si-TGF-β1 group compared with that in the si-NC group (Fig. 2a). To investigate the effect of TGF-β1 on the proliferation of endometriotic cyst stromal cells, cells were transfected with si-TGF-β1 or si-NC for 48 h, followed by CCK-8 and EdU assays. CCK-8 assay showed that downregulation of TGF-β1 obviously reduced the viability of endometriotic cyst stromal cells compared with control (Fig. 2b). In line with this, silencing of TGF-β1 markedly reduced the number of EdU-positive cells, as demonstrated by EdU assay (Fig. 2c).

### TGF-β1 knockdown inhibits the migration and invasion of endometriotic cyst stromal cells

**Fig. 3:**
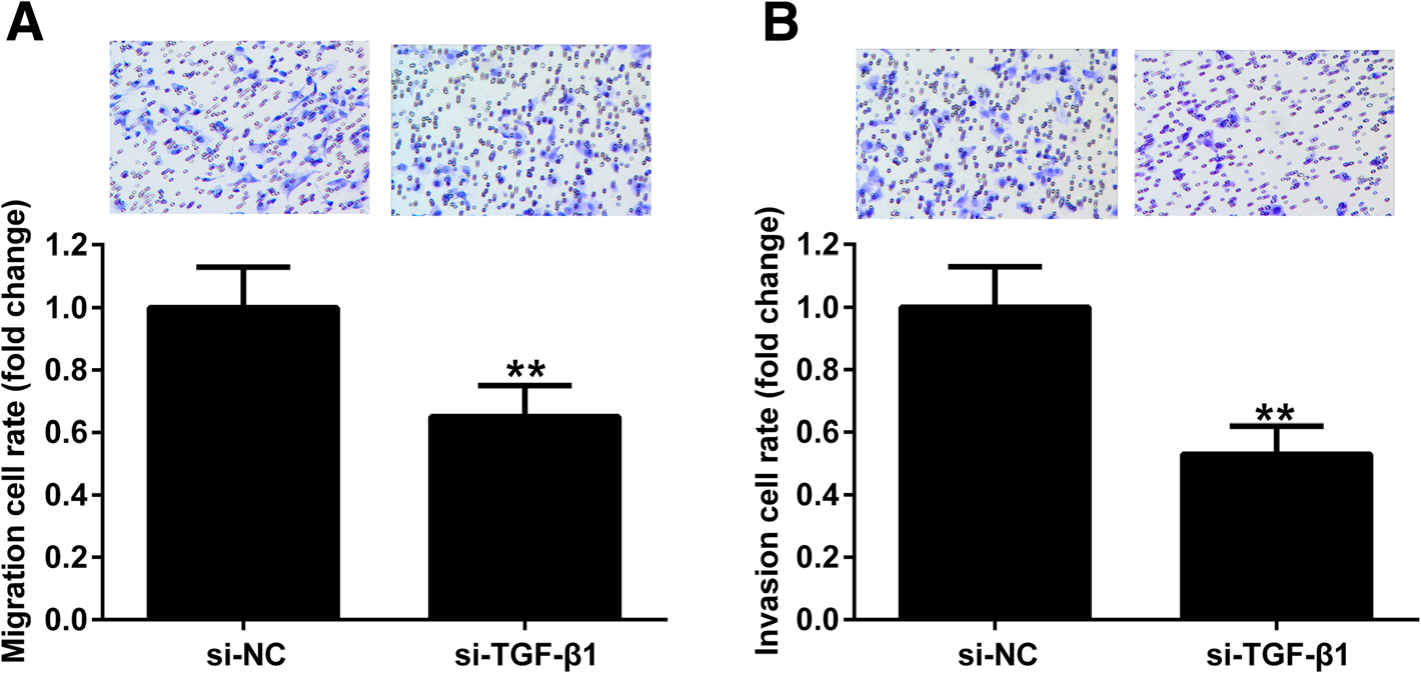
TGF-β1 knockdown inhibits the migration and invasion of endometriotic cyst stromal cells. Endometriotic cyst stromal cells were transfected with si-TGF-β1 or si-NC, and their migration and invasion abilities were assessed by transwell migration (**a**) and invasion (**b**) assays, respectively. **P < 0.01

To explore the effect of TGF-β1 on the migration and invasion of endometriotic cyst stromal cells, cells were transfected with si-TGF-β1 or si-NC for 48 h, and then subjected to transwell migration and invasion assays. As shown in Fig. 3a, silencing of TGF-β1 remarkably inhibited the migration of endometriotic cyst stromal cells. Meanwhile, silencing of TGF-β1 in endometriotic cyst stromal cells led to a marked decrease in cell invasion (Fig. 3b).

### TGF-β1 induces the proliferation, migration and invasion of endometriotic cyst stromal cells

**Fig. 4:**
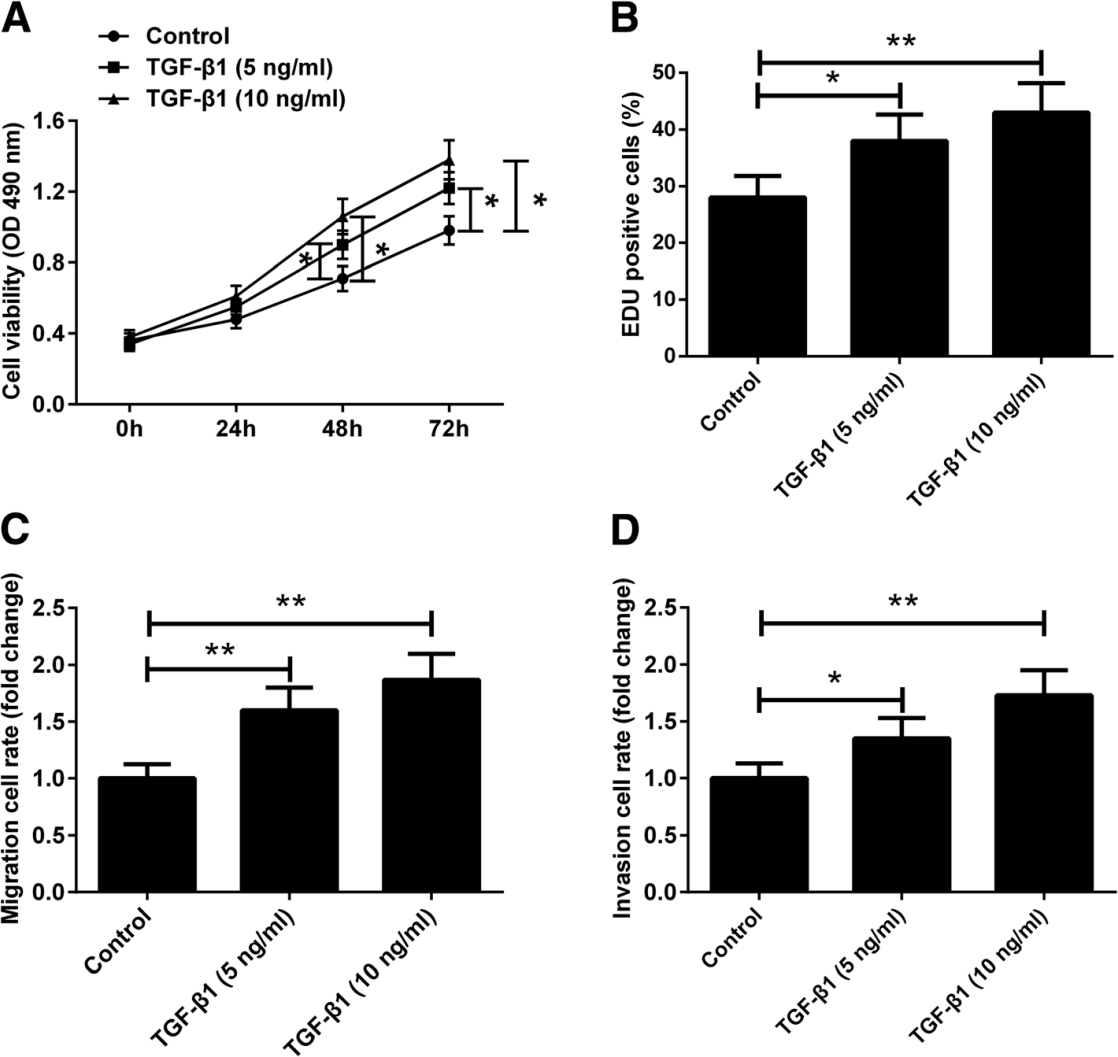
TGF-β1 treatment stimulates the proliferation, migration and invasion of endometriotic cyst stromal cells. Endometriotic cyst stromal cells were treated with recombinant human TGF-β1 (5 or 10 ng/mL) for indicated times (24, 48, or 72 h), and then subjected to CCK-8 assay (**a**). EdU assay was conducted to assess cell proliferation (**b**). The migration and invasion of endometriotic cyst stromal cells were assessed by transwell migration (**c**) and invasion (**d**) assays. *P < 0.05 and **P < 0.01

To validate the facilitating roles of TGF-β1 on the proliferation, migration and invasion of endometriotic cyst stromal cells, endometriotic cyst stromal cells were treated with recombinant human TGF-β1 (5 or 10 ng/ml). The results of CCK-8 assay and EdU assay showed that TGF-β1 treatment stimulated cell proliferation in a concentration dependent manner, as evidenced by increased cell viability and number of EdU positive cells (Fig. 4a and b). TGF-β1 treatment promoted the migration and invasion of endometriotic cyst stromal cells in a concentration dependent manner (Fig. 4c and d).

### TGF-β1-induced upregulation of VCAM-1 is dependent on Smad pathway

**Fig. 5:**
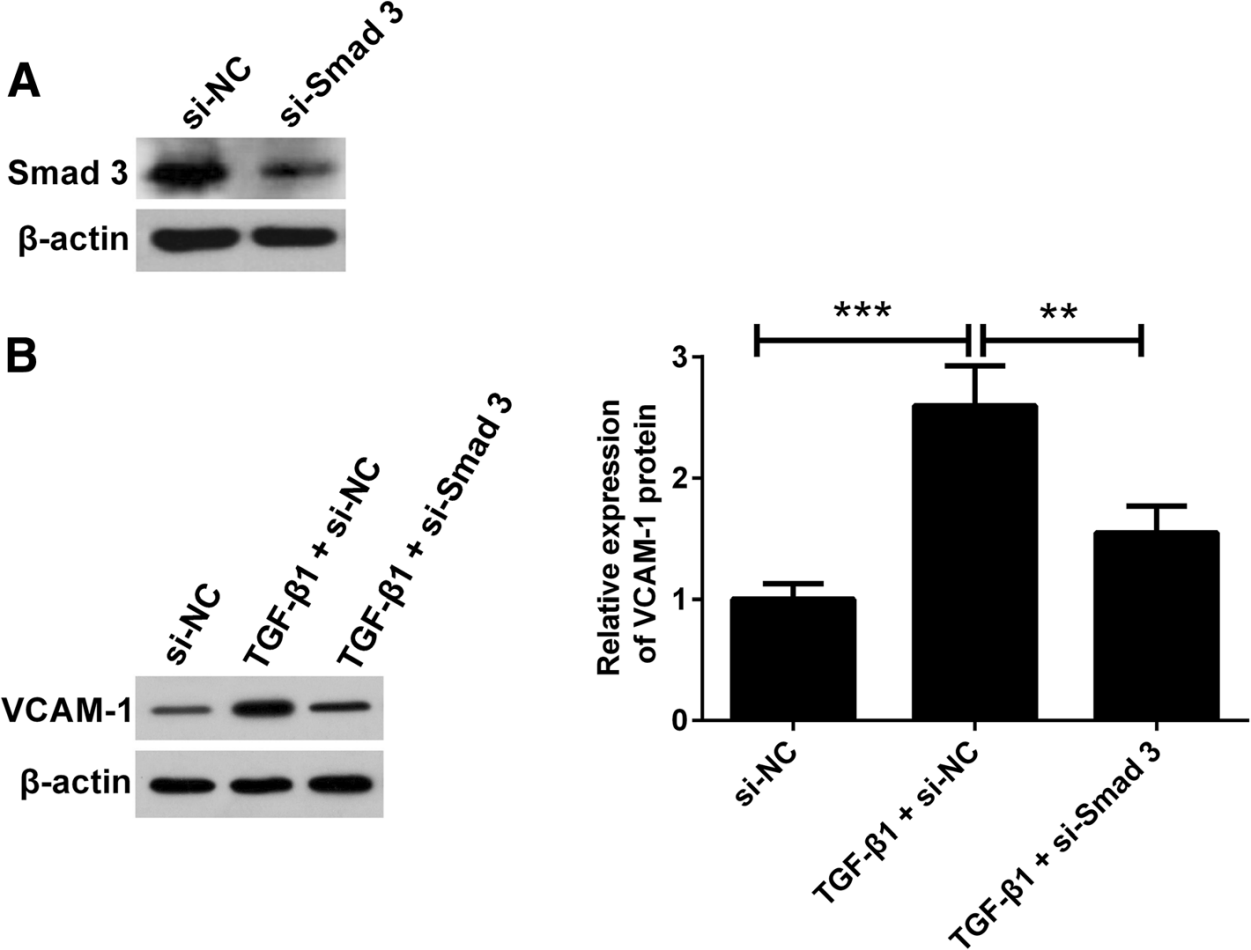
TGF-β1-stimulated upregulation of VCAM-1 is dependent on Smad pathway. The silence efficiency of si-Smad3 was confirmed by western blot assay (**a**). Endometriotic cyst stromal cells were transfected with si-Smad3, and then treated with recombinant human TGF-β1 (10 ng/mL) for 48 h. Cells were harvested for western blot assay (**b**). **P < 0.01 and ***P < 0.001

To determine Smad signal pathway induced by TGF-β1 is critical for stimulating VCAM-1 expression, we transfected endometriotic cyst stromal cells with si-Smad3 and then stimulated the cells with TGF-β1. The protein expression level of Smad3 was decreased after transfection of corresponding specific siRNA (Fig. 5a). In addition, knockdown of the expression of Smad3 abated the TGF-β1-stimulated VCAM-1 upregulation (Fig. 5b). These data suggested that the Smad pathway was involved in the regulation of the VCAM-1 expression stimulated by TGF-β1 in the endometriotic cyst stromal cells.

### Silencing of VCAM-1 blocks the effects of TGF-β1 on the proliferation, migration, and invasion of endometriotic cyst stromal cells

**Fig. 6:**
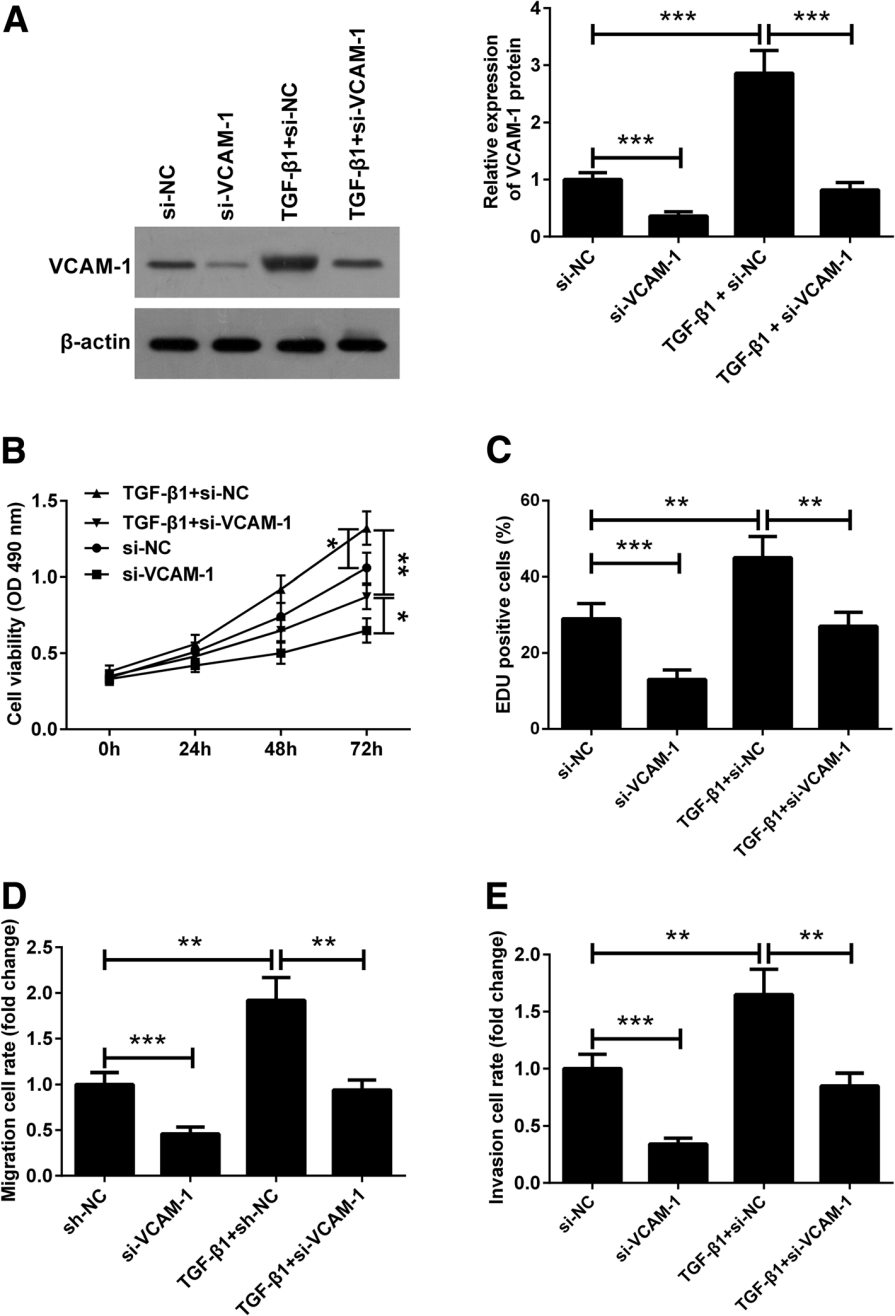
Silencing of VCAM-1 blocks the effects of TGF-β1 on the proliferation, migration, and invasion of endometriotic cyst stromal cells. Endometriotic cyst stromal cells were transfected with si-VCAM-1 or si-NC in the presence or absence of TGF-β1 (10 ng/mL) for different periods of time. **a** At 48 h post treatment, the protein expression of VCAM-1 was examined by western blotting. **b** and **c** The proliferation of endometriotic cyst stromal cells was determined by CCK-8 and EdU assays at the indicated time points. **d** and **e** At 48 h post treatment, the migration and invasion of endometriotic cyst stromal cells were determined by transwell migration and invasion assays. *P < 0.05, **P < 0.01, and ***P < 0.001

To further corroborate the role of VCAM-1 in mediating the effects of TGF-β1 on the proliferation, migration, and invasion of endometriotic cyst stromal cells, cells were transfected with si-VCAM-1 to silence VCAM-1 expression. Furthermore, the protein expression level of VCAM-1 was increased following TGF-β1 treatment, which was strongly reversed by silencing of VCAM-1 (Fig. 6a). The results of CCK-8 and EdU assays showed that transfection of endometriotic cyst stromal cells with si-VCAM-1 resulted in a significant decrease in cell proliferation compared to cells transfected with si-NC, as evidenced by reduced cell viability and decreased number of EdU-positive cells. Conversely, TGF-β1 treatment markedly increased the viability of endometriotic cyst stromal cells and elevated the number of EdU-positive cells. However, silencing of VCAM-1 remarkably blocked the promoting effect of TGF-β1 on endometriotic cyst stromal cell proliferation (Fig. 6b and c). In parallel, silencing of VCAM-1 inhibited the migration and invasion of endometriotic cyst stromal cells, while TGF-β1 treatment promoted the migration and invasion of these cells. More importantly, silencing of VCAM-1 could block the TGF-β1-induced promoting effects on cell migration and invasion in endometriotic cyst stromal cells (Fig. 6d and e). These data suggested that VCAM-1 played a critical role in TGF-β1-mediated proliferation, migration, and invasion of endometrial cells.

## Discussion

TGF-β, secreted by a variety of cells, is capable of triggering a shift in cell metabolism from mitochondrial oxidative phosphorylation to aerobic glycolysis, which is termed as the ‘Warburg effect’ [16]. In aerobic glycolysis, cells utilize glucose to produce a tremendous amount of lactate, which increases cell invasion [17]. Recently, increasing evidence supports the notion that TGF-β1 is a critical inducer of the occurrence and development of human endometriosis. For instance, TGF-β1 was found to be upregulated in endometriotic epithelial cells compared with that in normal endometrial cells [18]. TGF-β1 levels have been reported to be significantly elevated in the peritoneal fluid and serum of women with endometriosis, which may create a favorable environment for endometriotic lesion formation [19]. These researches suggested that ectopic expression of TGF-β1 was involved in the pathophysiology of endometriosis. Therefore, we further investigated the function and underlying mechanism of TGF-β1 in the regulation of proliferation, migration, and invasion of endometrial cells.

In a mouse model of endometriosis, TGF-β1-null mice exhibited a smaller endometriotic lesion compared with wild-type control mice, suggesting the involvement of TGF-β1 in endometriosis [20]. TGF-β1 has been shown to reduce the cytotoxicity of natural killer cells by downregulating the expression of NK Group 2, Member D in women with endometriosis [21], and was found to increase the expression of vascular endothelial growth factor-A through the inhibitor of DNA binding 1 pathway in women with endometriosis [22]. Additionally, TGF-β1 decreased the expression of c-fms and promoted the invasiveness of the immortalized endometrial epithelial cells [23]. Previously, TGF-β1 also enhanced the migration ability of endometriotic cells through upregulation of octamer-binding transcription factor 4 [24]. In accordance with these findings, our data showed that TGF-β1 expression was upregulated in endometriosis. In this study, we also explored the effect of TGF-β1 knockdown on the proliferation, migration, and invasion of endometriotic cyst stromal cells. With regard to the transwell migration and invasion assays, in order to reduce the influence caused by the cell proliferation and viability, endometriotic cyst stromal cells were resuspended in serum-free medium and then loaded into the upper chambers. For the invasion assay, transwell chambers were pre-coated with matrigel. Because the membrane pores were covered by matrigel, cells could not pass freely through the membrane pores. Invasive cells secrete hydrolase, at the same time, they also can easily pass through the membrane pores with matrigel, which is similar to the situation in vivo. Based on these experimental data, we demonstrated that knockdown of TGF-β1 inhibited the proliferation, migration, and invasion of endometriotic cyst stromal cells, indicating the pathogenic role of TGF-β1 in endometriosis.

VCAM-1 is a crucial cell surface adhesion molecule that belongs to the immunoglobulin superfamily. Evidence is now accumulating to suggest that the effects of TGF-β1 may be mediated by VCAM-1, which serves as a principal regulator of cell functions, such as cell proliferation, migration, and invasion. For instance, Agassandian et al. identified VCAM-1 as a TGF-β1-responsive mediator involved in idiopathic pulmonary fibrosis. They showed that TGF-β1 administration caused a significant increase in VCAM-1 gene transcription, which in turn promoted fibroblast cell proliferation [25]. Recently, it has been shown that VCAM-1 is a key player in regulating the behavior of endometriotic cells. VCAM-1 levels have been shown to be increased in the serum of women with endometriosis, which may be used as a potential diagnostic biomarker for endometriosis [26]. In this regard, TGF-β1 may regulate VCAM-1 expression to regulate the proliferation, migration, and invasion of endometriotic cyst stromal cells. The regulatory effect of TGF-β1 on VCAM-1 expression in endometriotic cyst stromal cells, confirmed by us in study, raises the possibility that TGF-β1 may promote the proliferation and invasion of endometriotic cells through VCAM-1. The significance of this study was to clarify the role of VCAM-1 in TGF-β1-mediated proliferation and invasion of endometriotic cells, thereby providing new insights into the pathological mechanism of endometriosis. Our findings suggest that the TGF-β1/Smad/VCAM-1 pathway is a vital inducer of the occurrence and development of endometriosis. Nevertheless, the present study has its own limitations. For instance, we did not detect the expression of TGF-β1 and VCAM-1 in eutopic endometrium of patients with endometriosis. The results of this study need to be further confirmed in animal models with endometriosis.

## Conclusion

We found that TGF-β1 and VCAM-1 expression levels were upregulated in the endometriotic tissues. Mechanically, knockdown of VCAM-1 inhibits TGF-β1-mediated proliferation, migration, and invasion of endometriotic cells. Our findings suggest that VCAM-1 may be a novel and effective therapeutic target for the treatment of endometriosis and offer scientific evidence for its clinical application. This study offers novel insights for understanding the pathogenesis of human endometriosis, thereby providing scientific evidence for exploring new treatments for human endometriosis.

## Data Availability

The datasets used and/or analyzed during the current study are available from the corresponding author on reasonable request.
